# High Terpene Production in Myrtaceae: Evolutionary Insights from Terpene Pathway Genes

**DOI:** 10.3390/plants15091293

**Published:** 2026-04-22

**Authors:** Xinlu Chen, Jin-Gui Chen, Gerald A. Tuskan, Feng Chen

**Affiliations:** 1 Department of Plant Sciences, University of Tennessee, Knoxville, TN 37996, USA; xchen24@utk.edu; 2 Center for Bioenergy Innovation, Oak Ridge National Laboratory, Oak Ridge, TN 37831, USA; chenj@ornl.org (J.-G.C.); tuskanga@ornl.gov (G.A.T.); 3 Biosciences Division, Oak Ridge National Laboratory, Oak Ridge, TN 37831, USA

**Keywords:** terpene pathway, evolution, Myrtaceae, Eucalyptus, gene expansion

## Abstract

Myrtaceae is one of the largest families of flowering plants and is well known for its prolific terpene production. To investigate the genetic basis underlying this high-level terpene output, we conducted comparative genomic analyses of genes of the entire terpene biosynthetic pathways in selected Myrtaceae species and representative species from three other families within the order Myrtales. Our analyses revealed that genes encoding enzymes in the upstream terpene biosynthetic pathways are generally conserved in copy number across Myrtales. Similarly, isoprenyl diphosphate synthases, which are positioned centrally and responsible for producing the direct precursors of major terpene classes, also exhibit conserved gene numbers among these species. In contrast, substantial differences were observed in the number of terpene synthase (*TPS*) genes, which function downstream and directly catalyze terpene formation. Myrtaceae species possess markedly more *TPS* genes than species from other Myrtales families. This expansion is primarily attributable to increased gene numbers in the TPS-a, TPS-b, TPS-g, and TPS-e/f subfamilies, with the first three subfamilies largely associated with sesquiterpene and monoterpene biosynthesis. Further analyses indicate that the enlarged TPS-a and TPS-g subfamilies resulted at the origination of Myrtaceae-specific groups, whereas TPS-b exhibited Myrtaceae-specific expansion. In *Eucalyptus grandis*, tandem duplication makes a larger contribution to the expansion of TPS-a, TPS-b and TPS-g subfamilies than interchromosomal duplication. The majority of these *TPS* genes exhibit moderate to high levels of expression in leaves, consistent with their role in elevated terpene production in leaves of *E. grandis*. Collectively, our findings are consistent with the hypothesis that the novel terpene-producing capacity of Myrtaceae is driven primarily by Myrtaceae-specific origination and/or expansion of downstream *TPS* genes rather than changes in upstream pathway gene copy numbers.

## 1. Introduction

Myrtaceae is an ecologically and economically important plant family comprising more than 6000 species [1], including globally important genera such as *Eucalyptus*, *Syzygium*, and *Psidium*. Members of this family dominate many tropical and subtropical ecosystems, where they play key roles in carbon sequestration, habitat structure, and fire-adapted landscapes [2,3,4,5]. Myrtaceae species are also major sources of fruit and timber, making them valuable for agriculture and forestry. In addition, Myrtaceae species produce rich phytochemicals [6,7]. These phytochemicals can be extracted as essential oils for various applications [8,9,10,11]. Among the diverse phytochemicals produced by Myrtaceae plants, terpenes are the best known [12,13,14]. Terpenes constitute the largest family of secondary metabolites produced by plants [15]. For Myrtaceae, they function in plant-environmental interactions [16,17], and because of their bioactivities, terpenes from Myrtaceae have many applications such as medicines, cosmetics, and biopesticides [18,19]. Due to their high energy density, terpenes synthesized by Myrtaceae species also represent promising feedstocks for advanced biofuels [20,21]. Despite their biological importance and applications, a family-wide assessment of the biosynthesis of terpenes in Myrtaceae has not been done.

**Figure 1:**
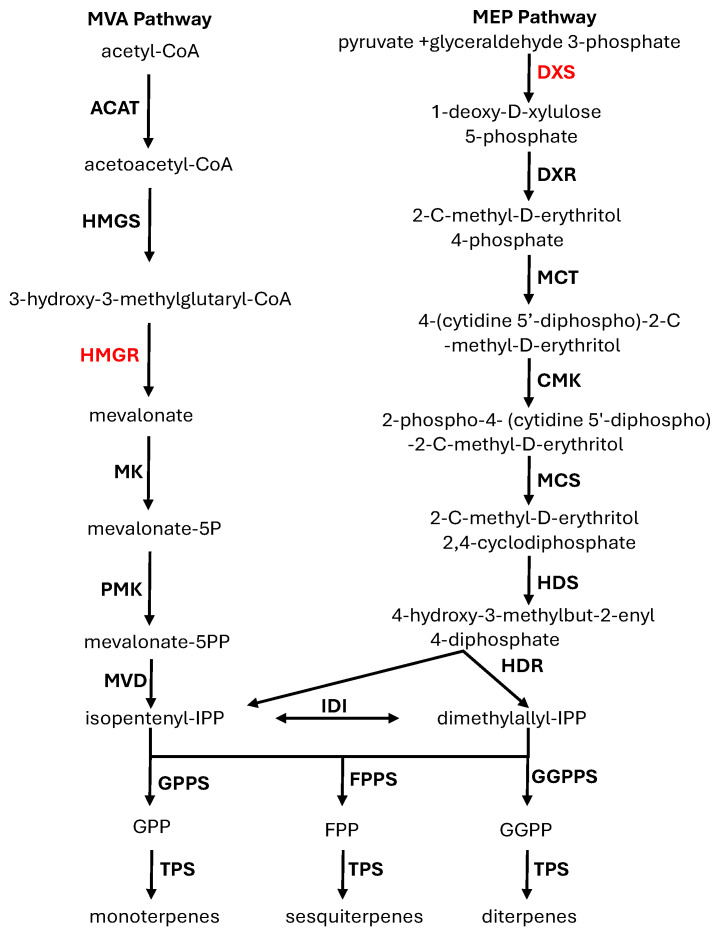
Terpene biosynthetic pathway and enzymes. The upper pathway includes the MVA pathway and MEP pathway. Enzymes of the MVA pathway include acetyl-CoA C-acetyltransferase (AACT), (3-3-methylglutaryl-CoA synthase (HMGS), 3-hydroxy-3-methylglutaryl-CoA reductase (HMGR), mevalonate kinase (MVK), phosphomevalonate kinase (PMK), mevalonate diphosphate decarboxylase (MVD), and isopentenyl diphosphate isomerase (IDI). Enzymes of the MEP pathway include 1-deoxy-D-xylulose 5-phosphate synthase (DXS), 1-deoxy- D-xylulose 5-phosphate reductoisomerase (DXR), 2-C-methyl-D-erythritol 4-phosphate cytidylyltransferase (MCT), 4-(cytidine 5-diphospho)-2-C-methyl-D-erythritol kinase (CMK), 2-C-methyl-D-erythritol 2, 4-cyclodiphosphate synthase (MCS), 4-hydroxy-3-methylbut-2-enyl diphosphate synthase (HDS) and 4-hydroxy-3-methylbut-2-enyl diphosphate reductase (HDR). HMGR and DXS, which are in red, are the rate-limiting enzyme of the MVA and MEP pathway respectively. The middle stage of the pathway is catalyzed by IDS, which include GPPS, FPPS and GGPPS. Their products are catalyzed by TPSs to form diverse terpenes.

Terpenes are synthesized through conserved three-stage biosynthetic pathways [15] that begin with the formation of the universal five-carbon building blocks isopentenyl diphosphate (IPP) and dimethylallyl diphosphate (DMAPP) via two parallel routes: the cytosolic mevalonate (MVA) pathway and the plastidial methylerythritol phosphate (MEP) pathway (Figure 1). In the second stage, chain-elongating isoprenyl diphosphate synthases [22,23] sequentially condense IPP and DMAPP to generate the primary terpene precursors geranyl diphosphate (GPP, C10), farnesyl diphosphate (FPP, C15), and geranylgeranyl diphosphate (GGPP, C20) (Figure 1). Finally, these isoprenyl diphosphates serve as substrates for terpene synthases (TPSs), which catalyze the committed and highly diversified cyclization and rearrangement reactions that produce the vast structural repertoire of monoterpenes, sesquiterpenes, and diterpenes (Figure 1) found across plants and other organisms [15,24].

Among terpene biosynthetic genes in Myrtaceae, a subset of *TPS* genes, primarily from the essential oil-rich genera *Eucalyptus* and *Melaleuca*, have been functionally characterized. Keszei, et al. (2010) [25] first reported TPS enzymes from Myrtaceae, showing that TPS1 from *Eucalyptus sideroxylon* produces multiple monoterpenes with 1,8-cineole as the major product, while TPS2 from *E. dives* produces β-caryophyllene. In *E. grandis*, several TPS enzymes have been biochemically validated and linked to major mono- and sesquiterpenes, supporting product predictions from genomic analyses [12]. For example, EgranTPS013 and EgranTPS041 both produce sesquiterpene mixtures, whereas EgranTPS059 yields multiple monoterpenes. Functional studies in *E. polybractea* further identified β-pinene and 1,8-cineole synthases [26]. In *Melaleuca alternifolia*, TPS enzymes have been directly linked to natural chemotypes, including linalool, sabinene/(Z)-sabinene hydrate, 1,8-cineole, and terpinolene synthases [27]. Collectively, these studies provide a growing set of experimentally validated TPS enzymes in Myrtaceae and begin to link TPS function with chemotype diversity.

Genomics is a powerful tool for uncovering the molecular mechanisms underlying diverse biological traits, including terpene biosynthesis. Genomic and comparative analyses have revealed that several Myrtaceae species possess expanded and highly dynamic repertoires of *TPS* genes. High-quality genome assemblies from species such as *Eucalyptus grandis* [28] show large *TPS* gene complements that are frequently organized in clusters, consistent with repeated tandem duplication events. This genomic expansion parallels the extraordinary terpene diversity that characterizes Myrtaceae foliage and essential oils. Myrtaceae belongs to the order Myrtales, which comprises nine families [1,29], and the number of sequenced species within this order continues to increase [30]. In contrast to Myrtaceae, plants from most other Myrtales families are not known for high-level terpene production. Thus, comparative genomic analyses of terpene biosynthetic pathway genes across Myrtaceae and related families may offer a unique opportunity to identify the molecular and genomic features associated with exceptionally high terpene production. In this study, we aim to conduct a comprehensive comparative genomic analysis of genes spanning the entire terpene biosynthetic pathways in Myrtaceae and related lineages to elucidate the genomic basis of high-level terpene production in this family.

## 2. Results and Discussion

### 2.1. Comparative Genomic Analysis of the MVA Pathway Genes

**Figure 2:**
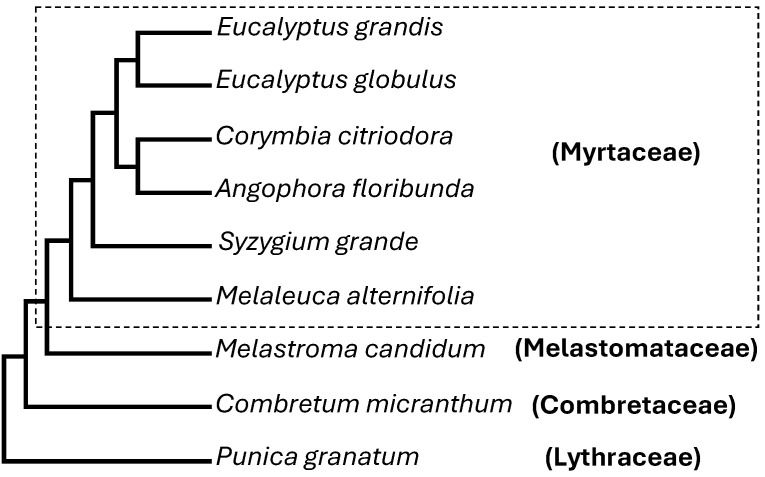
Phylogeny of the nine species analyzed in this study. The phylogeny was drawn according to [1,29]. These nine species belong to four families, which are indicated in parentheses.

With the growing list of species within Myrtaceae being sequenced, we selected six representative species including *E. grandis*, *E. globulus*, *Angophora floribunda*, *Melaleuca alternifolia*, *Corymbia citriodora*, and *Syzygium grande* (Figure 2) for comparative genomic analysis. For comparison within a broader evolutionary context, we selected one representative species from each of the three additional families within Myrtales, which include *Melastoma candidum* from Melastomataceae, *Combretum candidum* from Combretaceaem and *Punica granatum* from Lythraceae (Figure 2). While this multi-species comparison provides broad phylogenetic coverage, we acknowledge that the genomic datasets used were sourced from different databases and sequencing projects and therefore vary in assembly and annotation quality. Such differences may influence gene number counts and should be considered when interpreting cross-species comparisons. To minimize the potential impact of this limitation, we applied consistent criteria for gene identification across all species.

**Figure 3:**
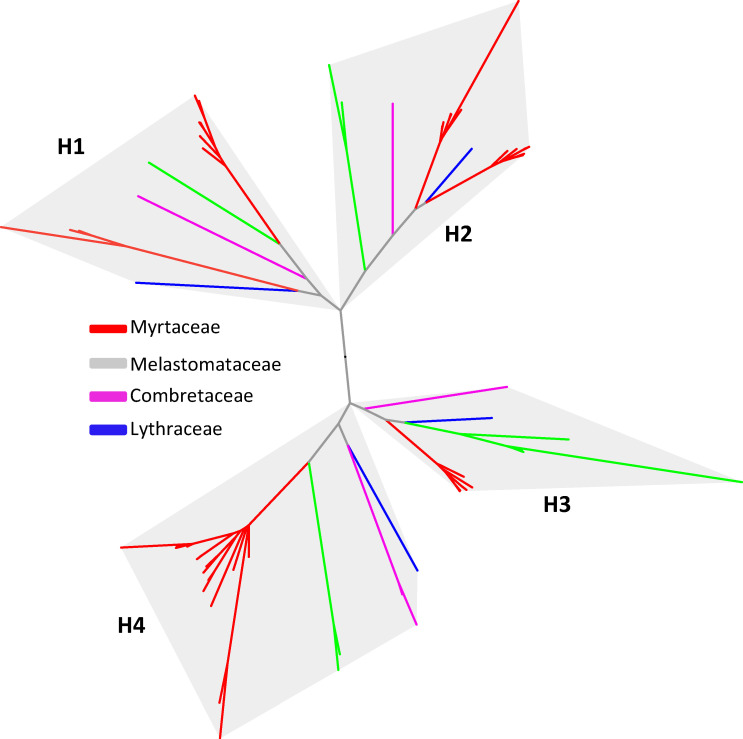
Phylogenetic tree of 3-hydroxy-3-methylglutaryl-CoA reductase (*HMGR*) genes from four families of Myrtales. A total of nine species listed in Figure 2 were analyzed. *HMGR* genes are color-coded according to their family origin. H1 to H4 indicate four clades, each of which shares a common ancestry among the four families.

The cytosolic MVA pathway contains seven enzymes including acetyl-CoA C-acetyltransferase (AACT), (3-3-methylglutaryl-CoA synthase (HMGS), 3-hydroxy-3-methylglutaryl-CoA reductase (HMGR), mevalonate kinase (MVK), phosphomevalonate kinase (PMK), mevalonate diphosphate decarboxylase (MVD), and isopentenyl diphosphate isomerase (IDI) (Figure 1). Genes encoding the seven enzymes of the MVA pathway in the genomes of the nine species were identified and compared. Most genes of the MVA pathway across the nine species showed overall conservation with limited variation in gene numbers, most with one or two copies (Table 1). In contrast, HMGR, which encodes the rate-limiting enzyme of the pathway [31], has a higher copy number in all species. Phylogenetic analysis of HMGR proteins resolved sequences into four clades (Figure 3), implying the preexistence of four copies of *HMGR* genes in the common ancestor of the four families of Myrtales analyzed. The copy number of *HMGR* genes across the four clades is largely conserved among species, with no apparent expansion in Myrtaceae. This stability in MVA pathway gene copy number, including HMGR, indicates that enhanced terpene production in Myrtaceae is unlikely to be driven by expansion of core MVA pathway genes.

### 2.2. Comparative Genomic Analysis of the MEP Pathway Genes

**Figure 4:**
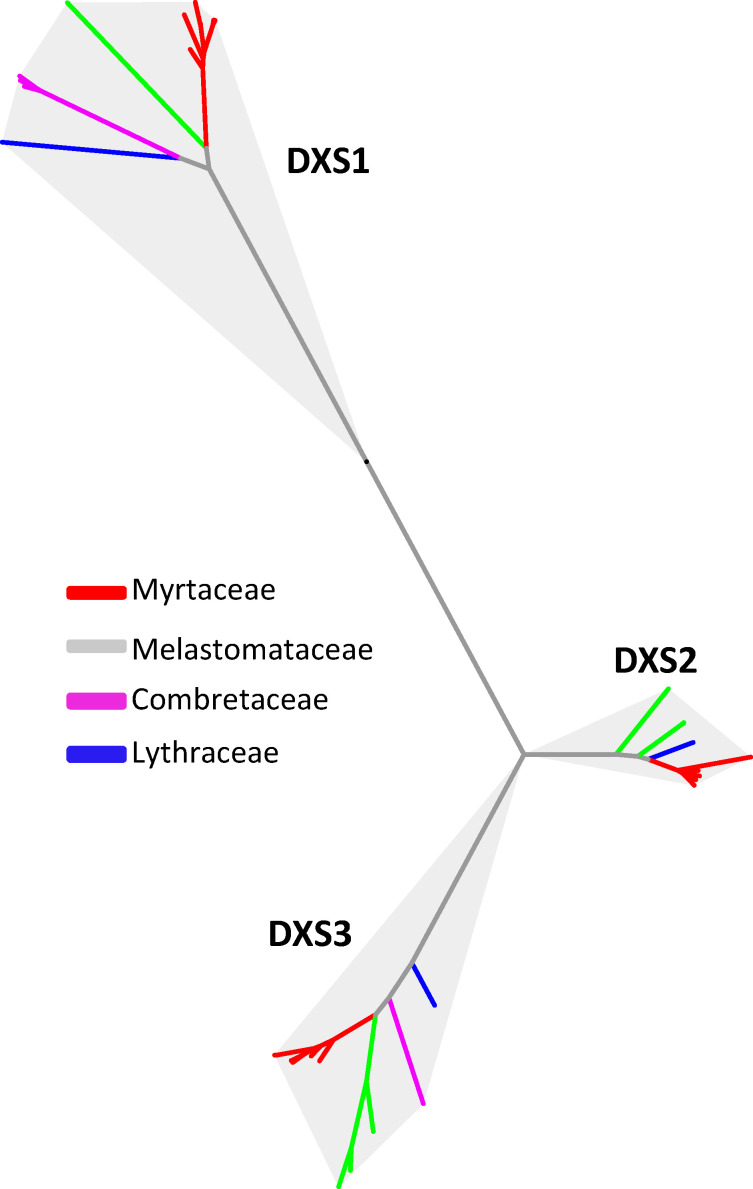
Phylogenetic tree of 1-deoxy-D-xylulose-5-phosphate synthase (*DXS*) genes from four families of Myrtales. A total of nine species listed in Figure 2 were analyzed. *DXS1*, *DXS2* and *DXS3* indicate three classes of *DXS* genes. *DXS* genes are color-coded according to their family origin.

The MEP pathway consists of seven enzymes that include 1-deoxy-D-xylulose 5-phosphate synthase (DXS), 1-deoxy- D-xylulose 5-phosphate reductoisomerase (DXR), 2-C-methyl-D-erythritol 4-phosphate cytidylyltransferase (MCT), 4-(cytidine 5-diphospho)-2-C-methyl-D-erythritol kinase (CMK), 2-C-methyl-D-erythritol 2, 4-cyclodiphosphate synthase (MCS), 4-hydroxy-3-methylbut-2-enyl diphosphate synthase (HDS) and 4-hydroxy-3-methylbut-2-enyl diphosphate reductase (HDR) (Figure 1). Genes encoding the seven enzymes of the MEP pathway in the genomes of the eight species were identified and compared. Most genes of the MEP pathway across the nine species showed overall conservation with limited variation in gene numbers, most with one or two copies (Table 2). In contrast, DXS, which is the first and rate-limiting enzyme of the MEP pathway, exhibited more pronounced copy number differences (Table 2). Phylogenetic analysis of DXS proteins from the nine species resolved sequences into three clades (Figure 4). This is consistent with the known observation that there are three classes of *DXS* genes, *DXS1*, *DXS2* and *DXS3*, in flowering plants [32]. In most of the nine species analyzed, each of the three DXS classes is represented by a single copy. Taken together, the conserved gene copy number of MEP pathway enzymes, including DXS, suggests that the elevated terpene production observed in Myrtaceae is unlikely to result from expansion of core MEP pathway genes.

### 2.3. Comparative Genomic Analysis of Isoprenyl Diphosphate (IDS) Genes

**Figure 5:**
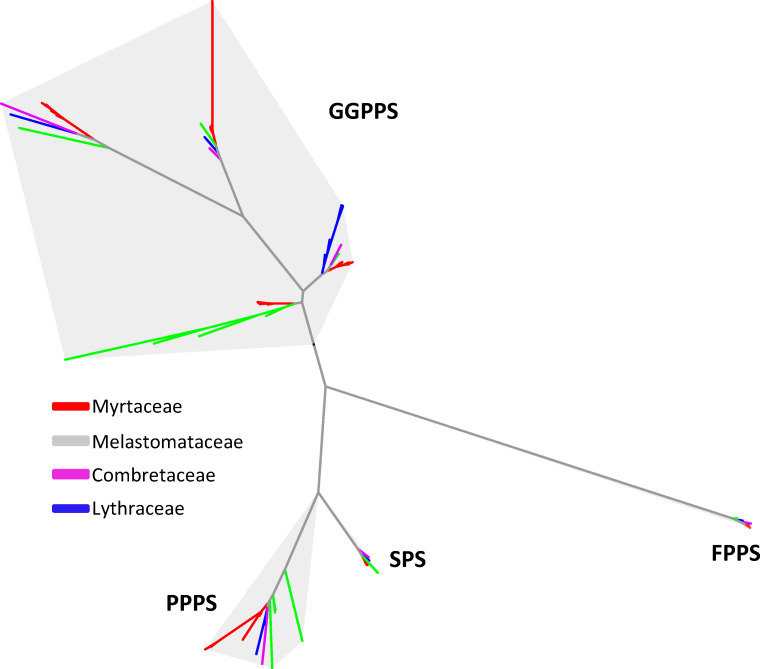
Phylogenetic tree of trans-isoprenyl diphosphate (*IDS*) genes from four families of Myrtales. A total of nine species listed in Figure 2 were analyzed. These genes belong to four subfamilies including *GGPPS*, *FPPS*, *SPS* and *PPPS*. *IDS* genes are color-coded according to their origin of family.

IDSs belong to four subfamilies, including the geranylgeranyl diphosphate synthase (GGPPS) subfamily, the farnesyl diphosphate synthase (FPPS) subfamily, the solanesyl diphosphate synthase (SPS) subfamily and the polyprenyl diphosphate (PPPS) subfamily [22,23]. While PPPS and SPS are involved in making long-chain isoprenyl diphosphates, GGPPS and FPPS are involved in making short-chain isoprenyl diphosphates, including GGPP and FPP. Phylogenetic analysis of *IDS* genes from the nine species of Myrtales resolved them into well-supported clades corresponding to four established subfamilies, FPPS, GGPPS, PPPS and SPS (Figure 5). All nine species contain a single *FPPS* gene. Most also contain a single *PPPS* and *SPS* gene (Table 3). In contrast, there is a large range in the number of *GGPPS* genes from five to 10 that diverge into four subclades. Despite this variation, the number of *GGPPS* genes from the six species of Myrtaceae are not significantly higher than those from the other three species/families. It should be noted that certain members of the GGPPS subfamily function as geranyl diphosphate synthases (GPPS) [33], and GPPS produces geranyl diphosphate, the direct precursor of monoterpenes, which are often the most abundant terpenes in Myrtaceae species. The conserved copy numbers of genes in the GGPPS and FPPS subfamilies suggest that subfamily expansion is unlikely to be the primary driver of the high-level production of monoterpenes and sesquiterpenes in Myrtaceae.

### 2.4. Comparative Genomic Analysis of TPS Genes

The TPS family is a large and functionally diverse group of enzymes responsible for generating the vast structural variety of plant terpenes [15]. Typical plant TPS genes fall into several well-defined subfamilies based on sequence similarity, domain architecture, and evolutionary history [15]. The TPS-a subfamily primarily contains sesquiterpene synthases that are common in angiosperms; TPS-b and TPS-g include monoterpene synthases and are enriched in specialized metabolism of flowering plants; TPS-c comprises ancestral, often diterpene-producing enzymes involved in primary metabolism; TPS-e/f includes diterpene synthases such as kaurene synthases essential for gibberellin biosynthesis [15,34], and TPS-d is a gymnosperm-specific clade that contains monoterpene synthases, sesquiterpene synthases and diterpene synthases [15,35]. These TPS subfamilies underpin the chemical and ecological diversity of plant terpene metabolism [15].

There are large differences in the number of *TPS* genes among the eight species. For the six species in Myrtaceae, the number of *TPS* genes in three species including *E. grandis*, *E. globulus*, and *C. citriodora* are over 100. There are 70 *TPS*s in *S. grande*, 83 *TPS*s in *M. alternifolia*, and 98 *TPS*s in *A. floribunda* (Table 4). The number of *TPS* genes in non-Myrtaceae species are much smaller with about 40 *TPS* genes in each of the three species. The higher sizes of the TPS family in species of Myrtaceae are consistent with previous observations [13]. The difference in the total number of *TPS* genes is related to the differences in the TPS-a, TPS-b, TPS-g and TPS-e/f subfamilies (Table 4).

**Figure 6:**
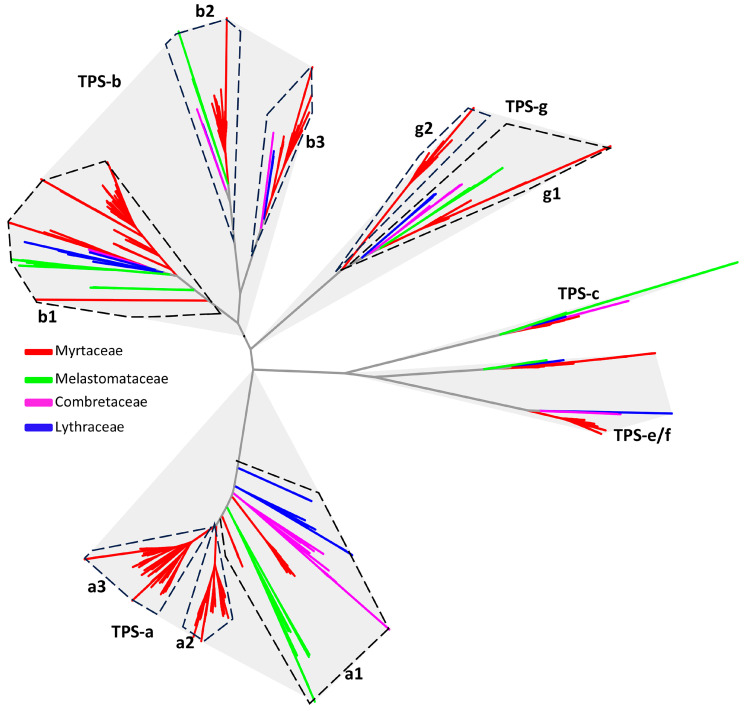
Phylogenetic tree of terpene synthase (*TPS*) genes from four families of Myrtales. A total of nine species listed in Figure 2 were analyzed. These genes belong to five subfamilies including the TPS-a, TPS-b, TPS-c, TPS-e/f and TPS-g subfamilies. Within TPS-a, TPS-b and TPS-g, specific clades are further defined. These specific clades and the TPS-c and -e/f subfamilies are indicated in dashed boundaries. TPS genes are color-coded according to their origin of family.

Phylogenetic analysis (Figure 6) resolved TPS proteins of Myrtales into well-supported clades corresponding to established TPS subfamilies, with clear separation of major lineages and evidence of both deep ancestral diversification and more recent, species-specific radiations. Within the TPS-a subfamily, genes clustered into three strongly supported clades, here designated as the TPS-a1, TPS-a2, and TPS-a3 groups (Figure 6). All nine species analyzed contained members of TPS-a2, whereas TPS-a2 and TPS-a3 were restricted to Myrtaceae (Table 5), suggesting family-specific diversification within this subfamily. Similarly, genes of the TPS-b subfamily formed three distinct clades, designated as theTPS-b1, TPS-b2, and TPS-b3 groups (Figure 6). TPS-b1 was present in all species, while TPS-b2 and TPS-b3 were detected only in Myrtaceae (Table 5). Notably, *M. candidum* lacked members of the TPS-b2 group. Genes of the TPS-g subfamily resolved into two well-supported clades, designated as the TPS-g1 and TPS-g2 groups (Figure 6). TPS-g1 was represented in all species, whereas TPS-g2 was exclusive to Myrtaceae (Table 5), consistent with lineage-specific expansion of this subfamily. In summary, phylogenetic analysis suggests that members of the Myrtaceae have evolved family-specific groups of *TPS* genes, e.g., TPS-a2, TPS-a3 and TPS-g2. In addition, all three groups in the TPS-b subfamily are expanded specifically in Myrtaceae. These newly derived and expanded *TPS* genes may provide the genetic basis of high-level production of monoterpenes and sesquiterpenes in Myrtaceae.

### 2.5. Distribution of Selected TPS Genes in the Genome of E. grandis

**Figure 7:**
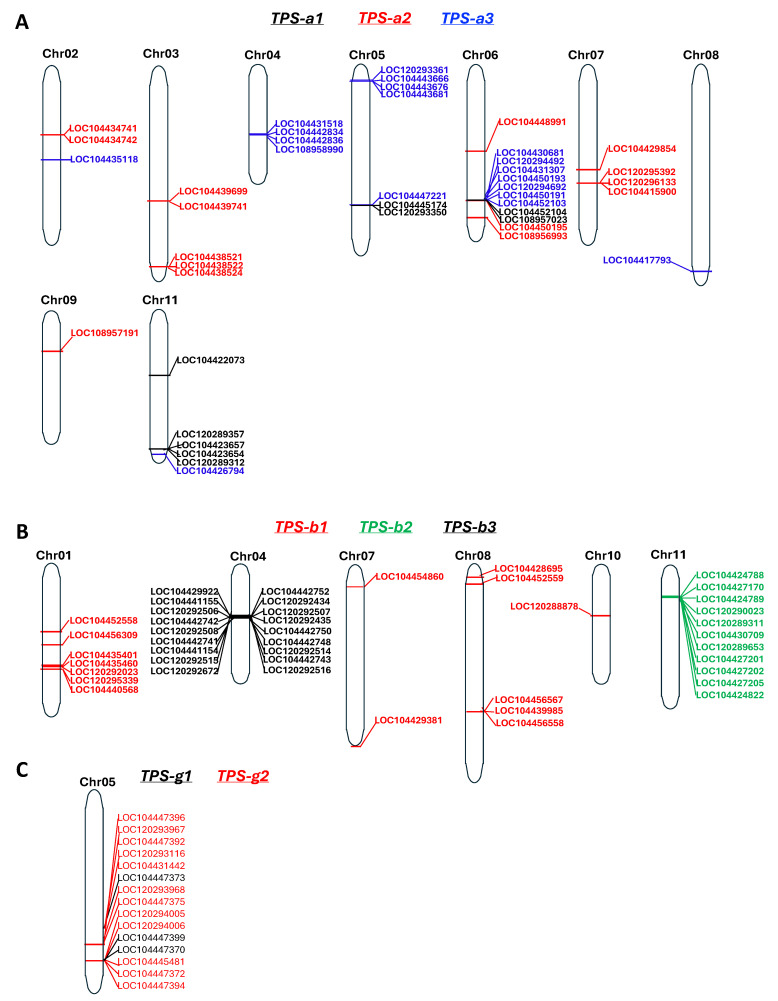
Chromosome location of selected terpene synthase (*TPS*) genes in *E. grandis*. (**A**) Location of genes of the of TPS-a1, TPS-a2 and TPS-a3 groups. Genes of the same group are the same color. (**B**) Location of genes of the TPS-b1, TPS-b2 and TPS-b3 group. Genes of the same group are the same color. (**C**) Location of genes of the TPS-g1 and TPS-g2 groups. Genes of the same group are the same color.

Chromosomal analyses of *TPS* genes in several Myrtaceae species have revealed that the expansion of this gene family occurred through both tandem duplications, in which gene copies are arranged consecutively on the same chromosome, and distal duplications, in which copies are located at separate chromosomal regions or on different chromosomes [12,13]. Here, we further examined the detailed chromosomal localization of *TPS* genes from those three major subfamilies, TPS-a, TPS-b, and TPS-g, with particular attention to the distribution of their respective phylogenetic groups. To this end, we chose *E. grandis* as the reference species, where the chromosomal location of the genes of the three subfamilies was determined. For the TPS-a subfamily (Figure 7A), TPS-a1 genes are distributed across three chromosomes with modest clustering, consistent with limited tandem duplication. TPS-a2 genes are localized on five chromosomes. TPS-a3 genes are distributed on six chromosomes. On chromosome 6, members of the three subclades are found in a cluster. In the other two locations, members of TPS-a1 and TPS-a3 form a cluster. The phylogenetic analysis suggests that TPS-a1 is ancestral and that TPS-a2/a3 are derived from gene duplication. The heterogeneous patterns of distribution of TPS-a1, TPS-a2 and TPS-a3 genes indicate a complex evolutionary history. Genes of the TPS-b subfamily are dispersed on seven chromosomes (Figure 7B). All of the 11 TPS-b2 genes are localized on chromosome 11 and they appear to be derived from tandem duplications. All 19 TPS-b3 genes are localized on chromosome 4. Similarly to TPS-b2, all TPS-b3 genes appear to be derived from multiple rounds of tandem duplications. In contrast, TPS-b1 genes are dispersed across five chromosomes, indicating alternate mechanisms underlying the evolution of TPS-b1 vs. TPS-b2 and TPS-b3 genes. Chromosomal mapping showed that both TPS-g1 and TPS-g2 genes are located on chromosome 5 but occupy two distinct loci. Rather than forming two separate clusters, TPS-g1 and TPS-g2 genes are intermingled (Figure 7C), suggesting an early gene duplication event through tandem duplication, specific to Myrtaceae and leading to the TPS-g1 and TPS-g2 groups, which was then followed by a segmental duplication leading to their occurrence on two separate chromosomes.

### 2.6. Expression Patterns of Selected TPS Genes in E. grandis

To further understand the functional evolution of genes in individual groups within the TPS-a, TPS-b, and TPS-g subfamilies, we examined and compared their expression patterns in *E. grandis* using publicly available transcriptomic datasets. Because leaves are the primary sites of terpene biosynthesis and storage, particular attention was given to gene expression in leaf tissue.

**Figure 8:**
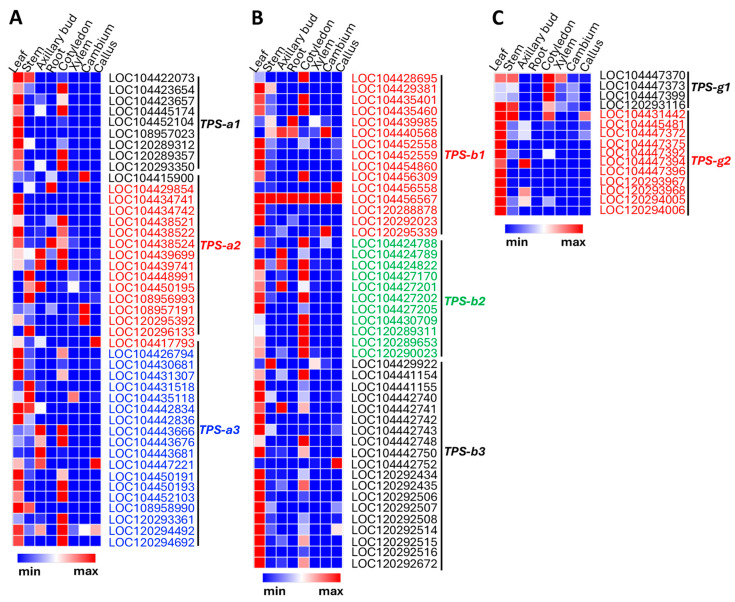
Expression of selected terpene synthase (*TPS*) genes in *E. grandis*. Expression data of individual TPS genes were extracted from EucaMOD [36]. (**A**) Expression pattern of TPS-a1, TPS-a2 and TPS-a3 groups in eight tissue types. Genes of the three groups are in different colors, consistent with those in Figure 7A. (**B**) Expression of TPS-b1, TPS-b2 and TPS-b3 groups in eight tissue types. Genes of the three groups are in different colors, consistent with those in Figure 7B. (**C**) Expression of TPS-g1 and TPS-g2 groups in eight tissue types. Genes of the two groups are in different colors, consistent with those in Figure 7C. “Min” and “Max” stand for the lowest and highest transcript levels, respectively.

For the TPS-a subfamily (Figure 8A), all ten genes in the TPS-a1 group of *E. grandis* showed high to moderate levels of expression in leaves, and six of them also displayed high expression in other tissues. In contrast, among the fifteen genes in the TPS-a2 group, only four showed high expression in leaves, whereas the remaining eleven were more highly expressed in other tissues. Genes in the TPS-a3 group exhibited patterns similar to those in TPS-a1, with eleven of the eighteen genes showing high expression in leaves with cotyledons being the next most prevalent tissue type. Overall, these patterns are consistent with the high accumulation of sesquiterpenes observed in leaves and suggest important contributions from the Myrtaceae-specific TPS-a2 and TPS-a3 groups.

For the TPS-b subfamily (Figure 8B), genes in the TPS-b1, TPS-b2, and TPS-b3 groups generally showed high expression levels in leaves. Among the fifteen TPS-b1 genes, ten exhibited high to moderate expression in leaves and six displayed leaf-specific expression. Notably, one gene (LOC104456567) showed high expression across all tissues examined. The TPS-b2 group contains eleven genes, eight of which showed high expression in leaves. Interestingly, eight TPS genes also showed high expression in cotyledons, including six that were highly expressed in leaves. Genes in the TPS-b3 group exhibited even stronger leaf dominance, with sixteen of the nineteen genes showing high expression in leaves. Overall, the expression patterns of TPS-b genes are consistent with the high accumulation of monoterpenes in leaves of *E. grandis*.

Genes of the TPS-g subfamily of *E. grandis* displayed a somewhat different expression pattern (Figure 8C). Among the four members of the TPS-g1 group, only two showed high or moderate expression in leaves, and these genes were also expressed at relatively high levels in other tissues. In contrast, all eleven members of the TPS-g2 group showed high expression in leaves but generally not elsewhere. This pattern suggests that the Myrtaceae-specific TPS-g2 genes contribute substantially to the high levels of monoterpene production in leaves.

## 3. Conclusions

Comparative genomic analyses across Myrtaceae and related families reveal that the core terpene precursor pathways (MVA and MEP) are largely conserved in gene numbers, with most enzymes present in one or two copies. In contrast, the rate-limiting enzymes HMGR (MVA pathway) and DXS (MEP pathway) exhibit lineage-specific expansions, suggesting that regulatory diversification of pathway entry points may contribute to metabolic specialization. Similarly, among *IDS* genes, *FPPS*, *PPPS*, and *SPS* remain mostly in conserved single-copy, whereas *GGPPS* has undergone substantial expansion in certain lineages, highlighting differential evolutionary pressures on chain-length-determining steps of terpene biosynthesis.

The most striking diversification occurs within the *TPS* gene family. Myrtaceae species possess dramatically expanded TPS repertoires compared with non-Myrtaceae relatives, primarily driven by subfamily-specific radiations in TPS-a, TPS-b, and TPS-g subfamilies. The conservation of upstream terpene pathway genes across Myrtaceae, combined with diversification of downstream *TPS* genes, suggests that terpene metabolic diversity in this family is driven primarily by adaptive evolution at the final biosynthetic steps rather than by innovation in core pathway genes. This pattern likely reflects a broader plant evolutionary strategy to maximize chemical diversity while maintaining metabolic efficiency. Phylogenetic and chromosomal analyses indicate that tandem duplication and segmental duplication have been the major mechanisms underlying this expansion, with distinct evolutionary trajectories among subclades. Myrtaceae-specific TPS groups, including TPS-a2, TPS-a3, and TPS-g2, show evidence of recent lineage-specific amplification and complex genomic rearrangements. Expression profiling further supports functional divergence among TPS subclades. Derived clades such as TPS-a3 and TPS-b1 exhibit stronger and more tissue-biased expression, particularly in foliar and vascular tissues associated with active terpene production, whereas ancestral or less-expanded clades show lower or more restricted expression. TPS-g1 and TPS-g2, though co-located on chromosome 5, display differential expression breadth, consistent with subfunctionalization following duplication. Together, these results demonstrate that while upstream terpene precursor pathways remain conserved, downstream terpene synthase genes have undergone extensive, lineage-specific expansion in Myrtaceae. This dynamic evolution of TPS genes likely underpins the remarkable chemical diversity and abundance of terpenes in this family.

Even though the copy numbers of genes in the MEP and MVA pathways and of *IDS* genes are generally conserved, it is possible that the expression levels of some of these genes are significantly higher in Myrtaceae, enabling greater metabolic flux. In addition, the presence of oil glands appears to provide a structural basis for the high-level biosynthesis and accumulation of terpenes in Myrtaceae [37]. This work establishes a foundation for testing the hypothesis that the abundant production of monoterpenes and sesquiterpenes characteristic of Myrtaceae is driven largely by family-specific origination of TPS-a and TPS-g subfamily genes and Myrtaceae-specific expansion of the TPS-b subfamily. The results presented here provide a genomic framework to guide future tissue- and cell-specific transcriptomic and proteomic investigations.

## 4. Material and Methods

### 4.1. Sequence Retrieval and Analysis

Proteomes of *E. globulus*, *C. citriodora*, *S. grande*, *M. candidum* and *P. granatum* were downloaded from NCBI (https:/www.ncbi.nlm.nih.gov/datasets/genome) (accessed on 1 October 2025). The protein dataset of *E. grandis* ANBG69807.140 was retrieved from EucaMOD [36] whereas the datasets for *C. micranthum* and *M. alternifolia* were obtained from Xie, et al. (2023) [38] and Voelker, et al. (2021) [39], respectively. A protein database for *A. floribunda* was not available. Therefore, its genome sequence was downloaded from NCBI.

### 4.2. Sequence Analysis

HMM profiles for terpene synthases (PF01397 and PF03936), trans-isoprenyl diphosphate synthases (PF00348), acetoacetyl-CoA thiolase (AACT; PF00108 and PF02803), 3-hydroxy-3-methylglutaryl-CoA synthase (HMGS; PF01154 and PF08540), 3-hydroxy-3-methylglutaryl-CoA reductase (HMGR; PF00368), mevalonate kinase (MK; PF00288 and PF08544), phosphomevalonate kinase (PMK; PF00288 and PF08544) and isopentenyl pyrophosphate (IDI; PF00293) were used to identify genes involved in the mevalonate (MVA) pathway, while HMM profiles for 1-deoxy-D-xylulose-5-phosphate synthase (DXS; PF13292), 1-deoxy-D-xylulose-5-phosphate reductoisomerase (DXR; PF02670), 2-C-methyl-D-erythritol 4-phosphate cytidylyltransferase (MCT; PF01128), 4-diphosphocytidyl-2-C-methyl-D-erythritol kinase (CMK; PF00288 and PF08544), 2-C-methyl-D-erythritol 2,4-cyclodiphosphate synthase (MDS; PF02542), 4-hydroxy-3-methylbut-2-enyl diphosphate synthase (HDS; PF04551), and 4-hydroxy-3-methylbut-2-enyl diphosphate reductase (HDR; PF02401) were employed to identify genes in the methylerythritol phosphate (MEP) pathway. All these HMM profiles were downloaded from Interpro (https://www.ebi.ac.uk/interpro/, accessed on 1 October 2025). All candidate genes were identified by Hmmsearch (https://www.ebi.ac.uk/Tools/hmmer/search/hmmsearch, accessed on 1 October 2025) with an e-value threshold of 1 × 10^−5^. Terpene pathway genes in *A. floribunda* were identified by performing BLAST 2.17.0 searches using terpene pathway genes from *E. grandis* as queries.

### 4.3. Phylogenetic Analyses

Multiple sequence alignments were performed using MAFFT version 7 [40] with 1000 iterations of refinement, using the E-INS-i algorithm for IDS, DXS, and HMGR and the L-INS-i algorithm for TPS. ProtTest 3 [41] was performed to select the most appropriate protein evolution model for each alignment under the Akaike Information Criterion. For the maximum likelihood analyses, RAxML version 8 [42] was used with 1000 bootstrap replicates under the best substitution models: JTT+G+F for TPS, and JTT+G for MGHR, DXS, and IDS. Phylogenetic trees were visualized using ITOL (https://itol.embl.de/, accessed on 1 October 2025).

### 4.4. Chromosomal Location and Expression Data of TPS Genes in E. grandis

The locations of selected *TPS* genes on chromosomes of *E. grandis* (ANBG69807.140) were created using MG2C v2.1 [43]. Expression data of selected TPS genes in *E. grandis* (ANBG69807.140) were downloaded from EucaMOD [36], and a heatmap was generated using the web-based interface of Morpheus (https://software.broadinstitute.org/morpheus, accessed on 1 October 2025).

## Figures and Tables

**Table 1:** Numbers of cytosolic mevalonate (MVA) pathway genes in the nine species of Myrtales.

Family	Species	*AACT*	*HMGS*	*HMGR*	*MK*	*PMK*	*MPDC*	*IDI*
Myrtaceae	*Eucalyptus grandis*	2	1	7	2	1	2	2
	*Eucalyptus globulus*	2	2	8	3	1	2	2
	*Angophora floribunda*	3	2	9	1	1	1	2
	*Corymbia citriodora*	2	2	8	2	1	2	2
	*Syzygium grande*	1	2	5	2	1	1	3
	*Melaleuca alternifolia*	2	4	7	2	1	3	2
Melastomataceae	*Melastoma candidum*	4	5	9	3	1	2	2
Combretaceae	*Combretum micranthum*	3	1	5	3	1	3	2
Lythraceae	*Punica granatum*	2	1	4	2	1	1	2

Abbreviations: acetyl-CoA C-acetyltransferase (AACT), (3-3-methylglutaryl-CoA synthase (HMGS), 3-hydroxy-3-methylglutaryl-CoA reductase (HMGR), mevalonate kinase (MVK), phosphomevalonate kinase (PMK), mevalonate diphosphate decarboxylase (MVD), and isopentenyl diphosphate isomerase (IDI).

**Table 2:** Numbers of plastidial methylerythritol phosphate (MEP) pathway genes in the nine species of Myrtales.

Family	Species	*DXS*	*DXR*	*MCT*	*CMK*	*MDS*	*HDS*	*HDR*
2Myrtaceae	*Eucalyptus grandis*	3	1	1	1	1	1	2
	*Eucalyptus globulus*	3	1	1	1	1	1	2
	*Angophora floribunda*	4	1	1	1	1	1	2
	*Corymbia citriodora*	4	1	1	1	2	1	2
	*Syzygium grande*	3	1	1	1	2	1	2
	*Melaleuca alternifolia*	3	1	1	1	1	1	1
Melastomataceae	*Melastoma candidum*	6	2	2	2	2	1	5
Combretaceae	*Combretum micranthum*	6	1	1	2	1	2	2
Lythraceae	*Punica granatum*	3	1	1	1	1	1	1

Abbreviations: 1-deoxy-D-xylulose 5-phosphate synthase (DXS), 1-deoxy- D-xylulose 5-phosphate reductoisomerase (DXR), 2-C-methyl-D-erythritol 4-phosphate cytidylyltransferase (MCT), 4-(cytidine 5-diphospho)-2-C-methyl-D-erythritol kinase (CMK), 2-C-methyl-D-erythritol 2, 4-cyclodiphosphate synthase (MCS), 4-hydroxy-3-methylbut-2-enyl diphosphate synthase (HDS) and 4-hydroxy-3-methylbut-2-enyl diphosphate reductase (HDR).

**Table 3:** Numbers of isoprenyl diphosphates genes in four subfamilies in the nine species of Myrtales.

Family	Species	*GGPPS*	*FPPS*	*PPPS*	*SPPS*
Myrtaceae	*Eucalyptus grandis*	5	1	4	1
	*Eucalyptus globulus*	6	1	1	1
	*Angophora floribunda*	7	1	2	1
	*Corymbia citriodora*	6	1	1	1
	*Syzygium grande*	5	1	1	1
	*Melaleuca alternifolia*	5	1	2	1
Melastomataceae	*Melastoma candidum*	10	2	4	2
Combretaceae	*Combretum micranthum*	5	1	2	1
Lythraceae	*Punica granatum*	7	1	2	1

Abbreviations: geranylgeranyl diphosphate synthase (GGPPS) subfamily, farnesyl diphosphate synthase (FPPS) subfamily, solanesyl diphosphate synthase (SPS) subfamily and polyprenyl diphosphate (PPPS) subfamily.

**Table 4:** Numbers of terpene synthases (*TPS*) genes in the nine species of Myrtales.

Family	Species	Total *TPS*	*TPS* Subfamilies				
			a	b	g	c	e/f
Myrtaceae	*Eucalyptus grandis*	112	43	45	15	3	6
	*Eucalyptus globulus*	113	46	37	12	2	16
	*Angophora floribunda*	98	42	33	10	2	11
	*Corymbia citriodora*	117	59	38	12	1	7
	*Syzygium grande*	70	29	24	9	1	7
	*Melaleuca alternifolia*	83	44	28	5	1	5
Melastomataceae	*Melastoma candidum*	42	15	15	6	4	2
Combretaceae	*Combretum micranthum*	44	24	5	9	2	4
Lythraceae	*Punica granatum*	44	17	19	5	1	2

**Table 5:** Numbers of genes in specific groups of terpene synthases (*TPS*) subfamilies in the nine species of Myrtales.

Family	Species	*TPS*-a			*TPS*-b			*TPS*-g	
		a1	a2	a3	b1	b2	b3	g1	g2
Myrtaceae	*Eucalyptus grandis*	9	15	19	15	19	11	4	11
	*Eucalyptus globulus*	7	16	23	20	10	7	1	11
	*Angophora floribunda*	6	23	12	17	7	9	6	4
	*Corymbia citriodora*	4	30	25	20	8	10	7	5
	*Syzygium grande*	7	12	10	15	5	4	4	4
	*Melaleuca alternifolia*	3	28	13	19	7	2	1	4
Melastomataceae	*Melastoma candidum*	15			12	3		6	
Combretaceae	*Combretum micranthum*	24			2	1	2	9	
Lythraceae	*Punica granatum*	17			15	1	3	5	

## Data Availability

The authors declare that all data supporting the findings of this study are available within the paper and its Supplementary Information Files.

## Supplementary Materials

The following supporting information can be downloaded at https://www.mdpi.com/article/10.3390/plants15091293/s1. Table S1. Number of *HMGR* genes in the four clades of the nine species of Myrtales.

## Notice

This manuscript has been authored by UT-Battelle, LLC, under Contract No. DE-AC05-00OR22725 with the U.S. Department of Energy. The United States Government retains and the publisher, by accepting the article for publication, acknowledges that the United States Government retains a non-exclusive, paid-up, irrevocable, worldwide license to publish or reproduce the published form of this manuscript, or allow others to do so, for United States Government purposes. The Department of Energy will provide public access to these results of federally sponsored research in accordance with the DOE Public Access Plan (http://energy.gov/downloads/doe-public-access-plan, accessed on 1 October 2025).
