# Supplementary material for: High Terpene Production in Myrtaceae: Evolutionary Insights from Terpene Pathway Genes
Source: Plants (Basel). 2026 Apr 22;15(9):1293. doi: 10.3390/plants15091293 (PMC13164918; doi:10.3390/plants15091293)
Supplement: Supplementary file 1 [file plants-15-01293-s001.zip › plants-4235325-supplementary.pdf]

Table S1. Number of *HMGR* genes in the four clades in the nine species of Myrtales

Family	Species	*HMGR* clades			
		H1	H2	H3	H4
Myrtaceae	*Eucalyptus grandis*	1	2	1	3
	Eucalyptus globulus	2	2	1	3
	Angophora floribunda	2	2	1	4
	Corymbia citriodora	2	2	1	3
	Syzygium grande	1	2	1	1
	Melaleuca alternifolia	2	2	2	2
Melastomataceae	*Melastoma candidum*	1	2	4	2
Combretaceae	*Combretum micranthum*	1	1	1	2
Lythraceae	*Punica granatum*	1	1	1	1
